# Assessing effective mechanical and chemical strategies for managing *Eucosma giganteana* (Lepidoptera: Tortricidae) in the perennial oilseed crop, *Silphium integrifolium* (Asteraceae: Heliantheae)

**DOI:** 10.1093/jisesa/iead102

**Published:** 2023-11-21

**Authors:** Ebony G Murrell, Konilo R Zio, Nervah E Chérémond, David L Van Tassel

**Affiliations:** The Land Institute, Salina, KS, USA; The Land Institute, Salina, KS, USA; Groupe ESA, Institute of Higher Education and Research in Life Sciences, Angers, France; Wooclap, Etterbeek, Belgium; The Land Institute, Salina, KS, USA; The Land Institute, Salina, KS, USA

**Keywords:** silflower, integrated pest management, perennial oilseed

## Abstract

*Eucosma giganteana* (Riley) is a native specialist pest of silflower, *Silphium integrifolium* Michx., which is currently being domesticated as a perennial oilseeds crop. The larvae of this moth attack silflower capitula and root crowns, causing both seed damage and long-term degradation of plants. To determine methods to manage *E. giganteana* in silflower crop fields, we conducted a laboratory bioassay and 3 field experiments to assess the effects of a suite of organic, conventional, and mechanical treatments on *E. giganteana* mortality and colonization of flower heads. Pyrethroids (permethrin, cyfluthrin), chlorantraniliprole, and methoxyfenozide each had significant insecticidal effects on *E. giganteana* in at least 2 of the experiments conducted. Nematodes marginally increased larva mortality in the laboratory bioassay and could be further investigated as a soil-applied biological control. In 2 separate field experiments, trimming the top 15% of silflower plants to delay flowering did not alone reduce *E. giganteana* colonization of flower heads throughout the growing season. However, when trimming was paired with a single chlorantraniliprole application, colonization of capitula was reduced by 83% over untreated control plants. Collectively, these experiments provide evidence for several treatments that could be further tested and incorporated into an integrated pest management strategy for *E. giganteana.*

## Introduction

The perennial sunflower *Silphium integrifolium* Michx., or silflower, is a native North American prairie plant that is being domesticated as a perennial oilseeds crop (Vilela et al. 2018). Like wild annual sunflower, silflower produces multiple yellow inflorescences and oily seeds; the oil composition of the seeds is similar to annual sunflower (Reinert et al. 2019). Unlike annual sunflower, silflower is a long-lived perennial with a woody root crown that produces new flowering stems every year after its establishment year. Once established, silflower is persistent under drought conditions (Price et al. 2022) and can live for more than 30 yr (Vilela et al. 2018). These qualities make silflower a prime candidate to domesticate as an oilseeds crop for growing regions where climate change has increased incidence of drought (Hudson et al. 2022).

Population buildup of specialist pests can be especially problematic in crop fields where the same crop is grown repeatedly, such as *Diabrotica virgifera* (LeConte) (Coleoptera: Chrysomelidae) in maize (Nelson et al. 1994), *Leptinotarsa decemlineata* (Say) (Coleoptera: Chrysomelidae) in potatoes (Casagrande 1987), and *Mayetiola destructor* (Say) (Diptera: Cecidomyiidae) in wheat (Clement et al. 2003). The perennial silflower is also subject to this phenomenon, as it is the host plant of the native herbivore *Eucosma giganteana*, which feeds only on plants in the *Silphium* genus (Johnson and Boe 2011, Vilela et al. 2018). Adult *E. giganteana* oviposit eggs on silflower flower heads as they begin to bloom in June–July. Neonate larvae burrow into the flower heads of *Silphium spp.* and feed until late summer. They then descend to the base of the plant and burrow into the root crowns, where they feed and overwinter as larvae before pupating and emerging the following summer (Johnson, et al. 2019).

**Fig. 1.**
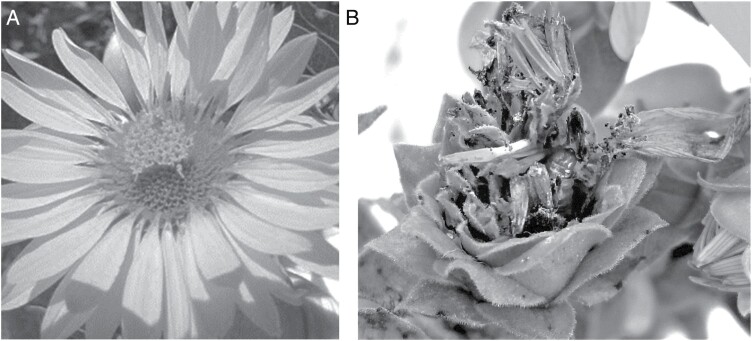
(A) a healthy silflower flower head in August (photo credit: David Van Tassel). (B) A silflower flower head at the same development stage, infested by an *E. giganteana* larva, visible in the lower right quadrant of the flower head (photo credit: Ebony Murrell).

**Fig. 2.**
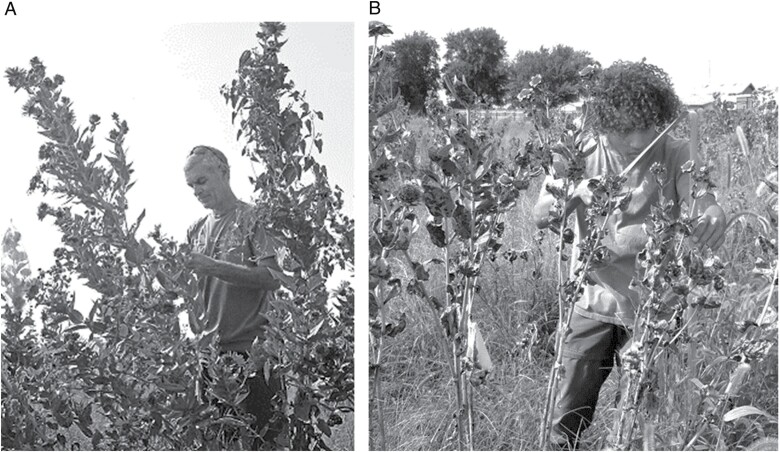
(A) A healthy silflower field in Salina, KS in summer 2017 at 2 yr old. (photo credit: David Van Tassel). (B) Silflower plants in the same field in summer 2019, after 3 yr of heavy *E. giganteana* infestation (photo credit: Ebony Murrell). The scientists in the 2 photos are within 7 cm height of each other.

The feeding patterns of *E. giganteana* larvae cause short- and long-term damage to silflower. Larval feeding on the flower heads directly damages developing seeds and induces flower rot (Fig. 1) (Prasifka et al. 2017). The autumnal tunneling of the larvae into the root crowns induces root rot and may reduce the carbohydrate stores of the plant. Established silflower plants typically do not die immediately, but show reduced height, biomass, and flower head production over multiple years of *E. giganteana* infestation (Fig. 2). Similar detrimental effects have been observed when *E. giganteana* feed on *Silphium perfoliatum* L. (Johnson et al. 2019), a close relative of silflower. Left unmanaged, *E. giganteana* infestations can reach 95% flower head colonization in silflower fields (Vilela et al. 2018). Given *E. giganteana*’s widespread geographic distribution in North America, from Minnesota south to Texas and east to Florida (Heppner 2003), and its negative effects on silflower’s viability and seed production, it is imperative to develop pest management strategies for this specialist pest if silflower is to be successfully domesticated and grown as a perennial oilseeds crop.

Since no insecticide is approved at the agronomic level to manage the highly specialized *E. giganteana*, the silflower breeding program had been using permethrin since 2012 to kill *E. giganteana* larvae inside flower heads that had been “bagged”, or enclosed in custom made cloth bags (Hubco, Inc, Hutchinson, KS). Bagging flower heads is necessary for the silflower breeding program to isolate flower heads from pollinators, and to add pollen from other genotypes to make specific plant crosses. Though less commonly used for control of Lepidoptera in North American field crops at this time, permethrin has been historically used to manage multiple lepidopteran species in field crops (Staetz 1985, Tabashnik et al. 1987, Abdelghafar et al. 1993). Two more recently developed insecticides could provide similar protection to developing silflower heads while reducing toxicity to nontarget species (e.g., natural enemies and pollinators). Methoxyfenozide is an ecdysone receptor agonist that alters apolysis in insect larvae, triggering premature molt in treated lepidopteran larvae and resulting in larval death (El-Sabrout et al. 2019). Chlorantraniliprole belongs to the insecticide class diamides, which are commonly used for management of lepidopteran pests in annual sunflower (Royer and Knodel 2019). Prior studies of the effects of these pesticides on tortricid moths in apple orchards (Grigg-Mcguffin et al. 2015) and cranberry bogs (Rodriguez-Saona et al. 2016) suggested that methoxyfenozide may have higher toxicity against tortricids than chlorantraniliprole. However, their efficacies in managing *E. giganteana* are currently unknown.

Multiple attributes of the silflower cropping system demand that an integrated pest management approach be developed for the management of *E. giganteana*. Though a recent meta-analysis indicates that specialist pest herbivores are less likely to develop resistance to a given insecticide than generalists (Hardy et al. 2018), those specialists that do develop insecticide resistance are among the most economically damaging. Examples include the Colorado potato beetle (*L. decemlineata*) in solanaceous crops, the diamondback moth (*Plutella xylostella* L., Lepidoptera: Plutellidae) in brassicas, and the western corn rootworm (*D. virgifera virgifera*) in corn (Meinke et al. 1998, Sparks and Nauen 2015). Therefore, it is prudent to develop a pest management strategy that does not solely rely on insecticides. It is also ideal to maintain whatever biological control may already help to manage a specialist pest. Though the ecology of *E. giganteana* is still poorly understood, there is evidence that it is fed upon by generalist predators (pers. obs.) and may also be infested by at least 1 parasitoid species (Hymenoptera: Braconidae) (Johnson et al. 2019). A third consideration is that silflower is an obligate cross-pollinated species and reliant upon a diverse array of pollinators to set seed (Prasifka et al. 2017). Therefore, any insecticides or practices used should minimally affect silflower pollinators or predator communities.

Sunflower pest pressure can sometimes be mitigated by planting seeds later in the season, such that the flowering time of the crop overlaps less with the peak emergence of sunflower pests (Prasifka et al. 2016). A similar effect can be achieved in perennials, such as alfalfa, by trimming or harvesting the plants to delay flowering to avoid seed pests (Summers 1998). Being adapted to ungulate grazing, silflower possesses the ability to regrow and flower when budding shoots are clipped (Vilela et al. 2020). Therefore, trimming silphium plants just prior to bloom could reduce *E. gigantea* colonization while still allowing the plant to flower and set seed later in the growing season.

To begin developing an integrated pest management strategy for *E. giganteana*, we conducted a series of laboratory and field experiments. We first determined the efficacy of a suite of conventional and organic insecticidal treatments on *E. giganteana* larvae in a laboratory bioassay. Next, we conducted a field experiment of a subset of these treatments on silflower stems naturally colonized with *E. giganteana* larvae. We then conducted 2 plot-sized experiments in which plants were either trimmed, or trimmed and sprayed once with a less broad-spectrum insecticide, to determine how mechanical manipulation alone versus a combination of trimming and insecticidal treatments affected the natural colonization of silflower plants by *E. giganteana*.

## Materials and Methods

### Field Site Description

All studies were conducted at The Land Institute (TLI) in Salina, KS, USA (38.768850, −97.566955). Both silflower and *E. giganteana* are native to this region and are commonly found in prairie remnants (Vilela et al. 2018). The breeding program for domestication of silflower as an oilseeds crop was initiated in 2002; however, the program underwent a significant increase in the size and numbers of silflower fields planted beginning in 2012 (Vilela et al. 2018). By 2017, abundance of *E. giganteana* in TLI silflower fields was measured at 95% flower head colonization by late July (Vilela et al. 2018).

**Fig. 3.**
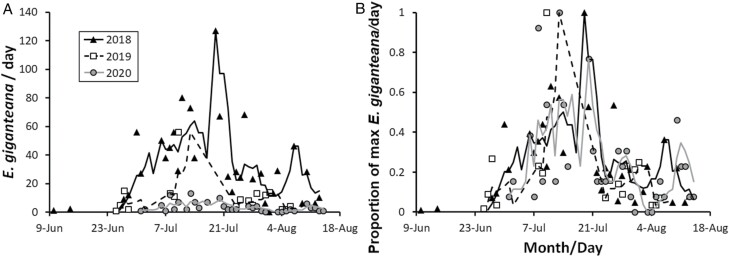
Daily records of *E. giganteana* collected from UV traps at The Land Institute, Salina, KS, USA between 2018 and 2020. (A) Raw data of daily capture rates. (B) Capture rates displayed as the proportion of the maximum daily capture within each year of collection. Lines represent the 4-day moving average of daily capture rates.

Beginning in 2018, 2 black light (UVA) light traps were annually set adjacent to semi-domesticated silflower fields on TLI property from June—August. The traps were made by the group. Each consisted of a 17-watt black light (HyperTough, Apex Tool Group, Sparks, MD) attached to a customized steel funnel with 4 steel vanes. The funnel was placed atop a custom-built 1.52 m^3^ screen cage. The trap lights were manually turned on at 5 pm and turned off at 8 am the following morning. Traps were checked daily for *E. giganteana* moths, and live moths were collected. Males and females were identified by the presence (males) or absence (females) of valvae at the distal end of the abdomen. The sexing criterion was confirmed by keeping moths alive in the laboratory after capture; moths identified via their genitalia as female laid eggs, while those identified as male did not. Once sexed, the number of moths of each sex was recorded. Days in which heavy rainfall occurred or the traps did not function were excluded from the dataset. Since no flight data have previously been recorded for *E. giganteana*, we utilized these local flight data (Fig. 3) to determine the timing of treatments in our subsequent field experiments.

### Laboratory Bioassay

To test for efficacy of potential insecticidal treatments, on 1 August 2018, we collected *E. giganteana* larvae from flower heads in a nontreated 4-yr old silflower field at The Land Institute, Salina, KS. Larger larvae (3 + instar) were placed in 59 ml plastic containers. Each container held ~25 ml of artificial diet. The diet is based on the recipe for artificial diet for *Homoeosma electellum* (Hulst) (Lepidoptera: Pyralidae) (Wilson 1990) with the following modifications: 75 g fresh silflower flower heads were added per liter diet, wheat germ was reduced to 10 g/liter diet, casein and sucrose were each reduced to 11.7 g/liter diet, and aureomycin was added at 0.37 g/liter diet to eliminate bacterial growth that otherwise would result from the introduction of fresh plant material. Adding aureomycin and incorporating silflower plant tissues in the diet were recommended by Dr. Jarrad Prasifka (Prasifka, pers. comm.). Other diet modifications were made by us in a preliminary study to rear *E. giganteana* on artificial diet, and resulted in *E. giganteana* successfully burrowing into and feeding on the diet. On 7 August, 168 larvae that had buried in the diet were randomized and assigned to 1 of 7 treatments (Table 1). Concentrations and application rates of the treatments were extrapolated from the recommended dosages recommended for lepidopteran pests on the labels, scaled down to the surface area of the plastic containers.

**Table 1.** List of organic and conventional insecticidal treatments used in the laboratory bioassay

Treatment	Treatment type	Active ingredient(s)	Amount of product/rep	Active ingredient/rep	ml H_2_O/rep	Equivalent field treatment
Altacor	Conventional	Chlorantraniliprole	0.06000 mg	0.02100 mg	0.18	4.5 oz/ac in 100 gal H_2_O
Baythroid XL	Conventional	β-Cyfluthrin	0.00004 ml	0.00001 ml	0.18	2.8 fl oz/ac in 100 gal H_2_O
PyGanic	Organic	Pyrethrins	0.00022 ml	0.00001 ml	0.36	15.61 oz/ac in 200 gal H_2_O
Monterey Bt	Organic	*Bacillus thuringiensis* subspecies *kurstaki(Btk)*	0.00121 ml	0.00119 ml	0.23	87.12 fl oz/ac in 130 gal H_2_O
Botanigard ES	Organic	*Beauvaria bassiana* strain GHA	0.00484 ml	0.00055 ml	0.18	348.48 fl oz/ac in 100 gal H_2_O
Triple Threat Nematodes (# Nematodes)	Organic	*Heterorhabditis bacteriaphora*, *Steinernema carpocapsae*, and *Steinernema feltiae*	~500 nematodes	~500 nematodes	0.18	136 million/ac in 12.22 gal H_2_O
Control	Not applicable	Not applicable	Not applicable	Not applicable	0.18	Not applicable

The diet in each container was perforated with 4 holes ~2 mm wide × 10 mm deep to improve the absorption of treatments into the diet. Treatments were mixed with RO water and applied to the diet surface in each container with a micropipette. The insecticide treatment solution was shaken every 4 replicates to ensure contents remained mixed. After treatment, larvae within each treatment were randomly assigned to 3 sampling dates: 1 day postapplication, 3 days, and 7 days. Cups were placed in the insect rearing chamber (26 °C, 14:10 L:D, 90% humidity).

At days 1, 3, and 7 posttreatment, the containers pre-assigned to these days were opened. Larvae were removed from the diet. Dead or moribund (alive, but nonfeeding and lethargic) larvae were assigned a value of 1 while live larvae were assigned a 0 value. We then analyzed the effects of fixed variables treatment and day, and the treatment × day interaction, on the proportion of dead/morbid larvae using general linear model analysis (PROC GLM SAS 9.4). Post hoc pairwise comparisons with a Tukey adjustment were used to determine differences in significant model effects, with α = 0.05.

### Field Stems Experiment

On 15 August 2018, we established a field study to test the extent to which different organic and conventional insecticidal treatments could mitigate damage in silflower flower heads caused by already-present *E. giganteana* larvae. This study compared the efficacy of tactics that had been previously employed by the silflower breeding program to eliminate *E. giganteana* from plants – covering flowering stems with bags (“bagging”) and spraying with a conventional insecticide (permethrin) – with 2 insecticides – *Bacillus thuringiensis* var. *kurstaki* (Bulla) (Bt*k*) and methoxyfenozide – that had not been previously tested on *E. giganteana* in the field.

The silflower field had been transplanted as seedling in 2016 as part of a breeding experiment. Plants were members of a breeding population that originated from wild populations in central Kansas but that had experienced several cycles of selection for domestication traits and some later introgressions from wild accessions obtained from other parts of the natural range of the species. Silflower plants were evenly spaced 91 cm apart from each other in a grid pattern. All plants were in full bloom at the time of the experiment. Sixty pairs of stems were selected on 13 plants, with 4–6 stem pairs per plant and 12–46 flower heads per stem pair. Pairs of stems were used to ensure sufficient flower heads were available for each replicate. Each stem pair had a minimum of 3 heads infested with *E. giganteana* and a minimum of 3 heads noninfested. We enclosed each stem pair in a single bag at the beginning of the study, and visibly infested flower heads were marked with pink fingernail polish. Colonization was assessed by the presence of a bore hole into the flower, a discolored stripe in the florets, and/or the presence of larva frass. Apart from marking infested flower heads with fingernail polish, the flower heads were left undisturbed for the duration of the study.

The insecticides selected for the field stems experiment were the organic insecticide Bt*k* (Monterey Bt, Sunlight Supply, Inc, Ontario, CA), and conventional insecticides permethrin (Gordon’s Permethrin 10, PBI/Gordon Corporation, Shawnee, KS) and methoxyfenozide (Intrepid 2F, Corteva Agriscience, Wilmington, DE). This insecticide was selected to be tested instead of chlorantraniliprole because it is a similarly narrow-range insecticide that has similar low toxicity to pollinators (Mommaerts and Smagghe 2011) and natural enemies (Kim et al. 2018) as chlorantraniliprole.

One of 6 spray treatments were applied, using a handheld spray bottle (Zep Chemically Resistant Sprayer, Zep, Inc., Atlanta, GA), to each stem pair: Control (sprayed with tap water, flowers left exposed), Bagged (sprayed with tap water and covered with a cloth bag), Methoxyfenozide (sprayed with 2.7 g Methoxyfenozide/955.5 ml water (0.28% concentration), flowers left exposed), Bagged + Methoxyfenozide (sprayed with 2.7 g methoxyfenozide/ 955.5 ml water (0.28% concentration) and bagged), Bagged + Btk (sprayed with 10.9 g Monterey Bt/946.6 ml water (1.15% concentration) and bagged), or Bagged + Permethrin (sprayed with 3.0 g Permethrin/ 973 ml (0.30% concentration) and bagged). The bag on each stem was rolled up over the flower, and 10 sprays were applied inside the bag (~12 ml) such that all flowers were wetted. The permethrin application rate was the equivalent of 425 g/ha. Though this is higher than the rate recommended for *Cydia pomonella* (a tortricid, like *E. giganteana*) in apple orchards (280 g/ha), the rate used was in keeping with the application method previously used by the silflower breeding program. The methoxyfenozide application rate was the equivalent of 425 g/ha, the maximum application rate recommended by the pesticide label for treating *C. pomonella* in apple orchards. The bags were then rolled down and secured to the stem underneath the sprayed flower heads for the flower-exposed treatments (Control and Methoxyfenozide); otherwise, bags were folded over at the top and secured with a clothespin for all other treatments. Bagging stems allowed us to assess the damage of already-present larvae without risk of further oviposition by adults, nor migration of the larvae to the root crown. The unbagged treatments were used to assess the degree to which oviposition/larva migration could affect the results observed with no treatment (Control) or the application of a relatively low toxicity insecticide (Methoxyfenozide).

After 16 days, we removed the stem pairs from the field and dissected them in the laboratory to determine the number of live *E. giganteana* larvae per stem pair. Seeds were threshed by hand and sorted into filled (mature achene felt when hull was pressed with a finger), unfilled (no detectable achene in hull), and damaged (visible signs of chewing on the hull).

We used linear mixed-model analyses (PROC MIXED, SAS 9.4) to test whether the number of larvae at study termination, the proportion of damaged achenes, the proportion of filled achenes, and the proportion of unfilled achenes were affected by the covariate of the proportion of flower heads initially colonized, by the fixed effect of treatment, and by the interaction between proportion of flower heads initially colonized and treatment. The plant on which the stem pairs resided was also included as a random effect. Differences between significant effects in each model were analyzed using post hoc pairwise comparisons with a Tukey-Kramer adjustment.

### Trimming + Insecticide Field Plot Experiments

The 3 fields used for this experiment in 2019 and 2020 were located at The Land Institute, Salina, KS, USA (“East Bank”: 38.771083, −97.569692; “Farm North”: 38.770889, −7.592667 and “Farm South”: 38.770222, −97.593028). All fields had been transplanted as seedlings in 2016 in 91 cm rows. Plants were a mixture of semi-domesticated and wild silflower accessions. Each field was divided into 4 plots: (i) A control, in which plants were neither trimmed nor sprayed, (ii) “Trim”, in which the top 15% of the plants were trimmed using a hedge trimmer before the flower heads matured, (iii) “Trim+Early Spray”, in which plants were trimmed as in Treatment 2, then also sprayed with an insecticide in early July when the adult *E. giganteana* were in early flight, (iv) “Trim+Late Spray”, in which plants were trimmed as in Treatment 2, then sprayed with an insecticide in late July when the adult *E. giganteana* were at or near peak flight. The same 4 treatments were applied to the same plots and fields in both years, with only the insecticide used differing in the trim + spray plots in 2019 and 2020. Spray treatments were applied using a handheld 45 PSI pump sprayer (GroundWorks, Tractor Supply, Brentwood, TN).

In 2019, Treatments 2–4 were trimmed in the East Bank and Farm South fields on 5 May; the Farm North field was trimmed on 22 May due to the slower development of the plants in that field. The insecticide methoxyfenozide (Intrepid) was applied to the flower heads of the Trim + Early Spray plots on 2 July, using a pump sprayer, at the rate of 132 g/ha. The same insecticide was applied at the same rate to the Trim + Late Spray plots on 26 July.

On July 11, 5 plants were randomly selected and flagged in each plot. The terminal flower head of each stem was checked biweekly for signs of colonization, as described in the field stems experiment. Stems in which the terminal flower head was colonized were marked with flagging tape. The weekly data collected per plant from 11 July 11 to 4 September included the number of stems per plant (new stems can grow throughout the summer, and stems can also be lost to rot or mechanical weeding), the number of stems newly colonized, and the total number of colonized stems.

In 2020 the sprayed treatments were sprayed using chlorantraniliprole (Prevathon, Corteva Agriscience, Wilmington, DE) at the rate of 58 g/ha, instead of the methoxyfenozide used in 2019. This rate is slightly less than the 61.7 g/ha application rate recommended for use of chlorantraniliprole (Altacor, Corteva Agriscience, Wilmington, DE) on *C. pomonella* in apple orchards. Trim + Early Spray plots were treated on 2 July, and Trim + Late Spray plots were treated on 19 July. Five plants per plot were flagged, and number of stems, new colonization per stem, and total stems colonized were assessed weekly from 8 July to 28 August using the same methods as in 2019. However, in 2020 *E. giganteana* populations were much lower, likely due to the implementation of new management practices in neighboring silflower fields in fall 2019. Therefore, the criterion for “stem colonization” was that only 1 of any flower head on a stem showed signs of *E. giganteana* colonization, not necessarily the terminal flower head.

We used repeated-measures mixed models (PROC MIXED SAS 9.4) for each study to analyze whether the proportion stems colonized changed as a function of sample week, treatment (both fixed effects), or the interaction of week × treatment. In this model week was the repeated-measures variable, with individual plot as the subject, and the AR(1) covariance structure was used. Study field was included as a random effect. The proportion colonization data from 2019 was square-root transformed to meet the assumption of normality. Significant differences in the mixed models were further analyzed using post hoc pairwise comparisons with a Tukey–Kramer adjustment.

## Results

### Laboratory Bioassay

**Fig. 4.**
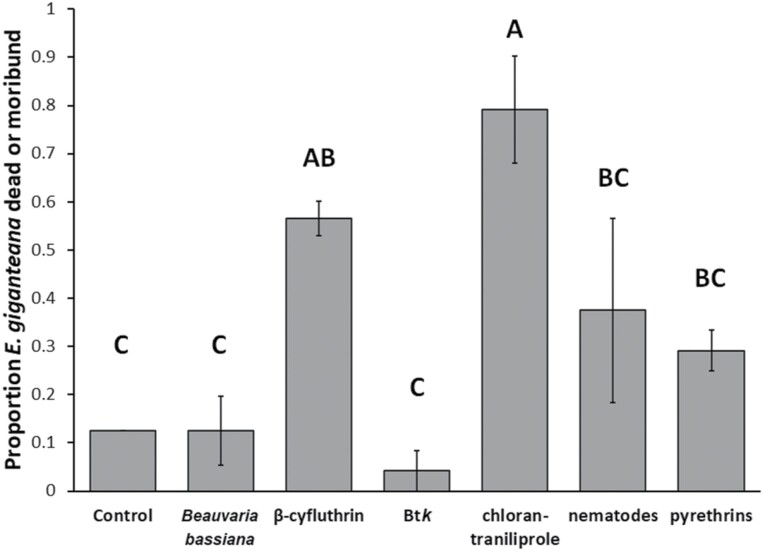
Mean ± SE proportion *E. giganteana* larva death and morbidity in the laboratory bioassay of insecticidal treatments. Means with shared letters do not significantly differ from one another (α = 0.05).

The proportion of dead or moribund larvae posttreatment significantly differed by treatment (*F* = 10.81, df = 6, 146, *P* < 0.0001) (Fig. 4). Btk, *Beauvaria bassiana*, pyrethrins, and control did not significantly differ from one another. Nematodes performed arithmetically, though not significantly, better than Bt*k* (*P *= 0.0684); nematodes also did not outperform *B. bassiana* or control. Both chlorantraniliprole and β-cyfluthrin significantly outperformed control, *B. bassiana*, and Bt*k*, and chlorantraniliprole also outperformed nematodes and pyrethrins. There was no significant effect of days posttreatment (*F* = 1.75, df = 2, 146, *P* = 0.1767), and no significant treatment × day interaction effect (*F* = 1.16, df = 12, 146, *P* = 0.3208).

### Field Stems Experiment

**Fig. 5.**
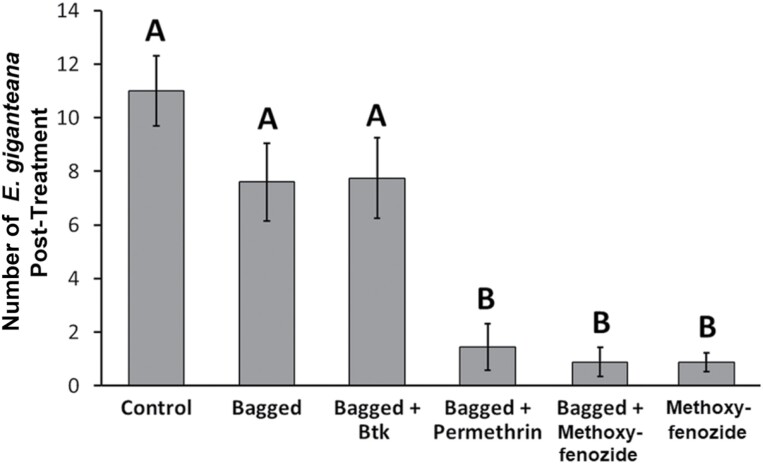
Mean ± SE *E. giganteana* per replicate of field stems. Means with shared letters do not significantly differ from one another (α = 0.05).

For all mixed models in Table 2, the interaction between proportion of initial flower heads colonized and treatment were not significant (data not shown); therefore, the interactions were removed from these models to increase statistical power to test main effects. There was a significant difference in the number of live *E. giganteana* across treatments 16 days postapplication (Table 2). Bagging alone did not reduce the final number of *E. giganteana* larvae compared to the unbagged control, nor did the application of Btk on bagged stems (Fig. 5). However, larva abundance was significantly lower on bagged stems sprayed with permethrin, and on stems sprayed with methoxyfenozide whether bagged or unbagged (Fig. 5). There was also a significant negative effect of the covariate of the initial number of flower heads colonized prior to treatment application on the number of larvae per stem pair posttreatment (slope = −10.2434 ± 3.4377).

**Table 2.** Mixed model results for the 2018 field stems experiment: number of larvae 16 days posttreatment, proportion of damaged achenes, proportion of achenes filled, and proportion of achenes unfilled. *P < 0.05, **P < 0.01, ***P < 0.001

Model parameter	# Larvae posttreatment			Proportion damaged achenes			Proportion filled achenes			Proportion unfilled achenes		
	*F*	df	*P*	*F*	df	*P*	*F*	df	*P*	*F*	df	*P*
Treatment	22.08	5,37	<0.0001***	4.29	5,29	0.0049**	1.62	5,29	0.1868	0.89	5,29	0.5019
Proportion capitula initially infested	8.88	1,37	0.0051**	7.42	1,29	0.0108*	0.06	1,29	0.8090	2.65	1,29	0.1144
Plant covariance	1.6413			0.0055			0.0141			0.0152		
Residual covariance	7.6059			0.0038			0.0095			0.0132		

**Fig. 6.**
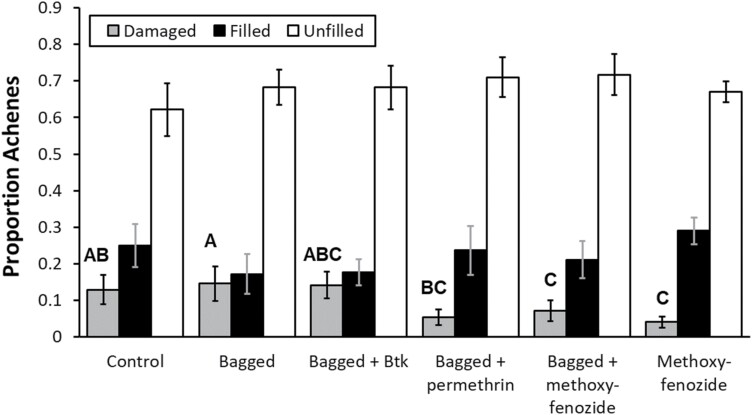
Least squared mean + SE of proportion achenes filled, unfilled, or damaged in the field stems experiment. Means with shared letters for proportion achenes damaged do not significantly differ from one another (α = 0.05).

The proportion of achenes damaged per flower head significantly differed by treatment (Table 2). Stems with methoxyfenozide treatments, whether bagged or unbagged, had a lower proportion seeds damaged than control plants (Fig. 6). There was also a positive effect of initial proportion heads colonized on the proportion of achenes damaged (0.2418 ± 0.0888). Neither the proportion of filled achenes nor the proportion of unfilled achenes were significantly affected by treatment or the proportion of flower heads colonized prior to treatment applications (Table 2, Fig. 6).

### Trimming + Spray Field Plot Experiments

**Fig. 7.**
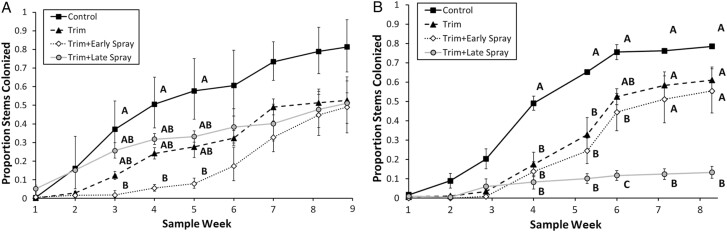
Mean ± SE proportion of silflower stems colonized by *E. giganteana* larvae in the 2019 and 2020 field experiments. (A) The 2019 field experiment, which used the insecticide methoxyfenozide in spray treatments. (B) The 2020 field experiment, which used the insecticide chlorantraniliprole in spray treatments. Shared letters within sample dates indicate treatments that do not significantly differ from each other (α = 0.05). Only sample dates in which there were significant differences between treatments are lettered.

In 2019, when methoxyfenozide was applied as the insecticide, there was a significant interaction between sample week and treatment (Table 3). The “Trim+Early Spray” significantly reduced the proportion of colonized stems in comparison to the control group in sample weeks 3–5 (Fig. 7A). However, those 2 treatments became statistically indistinct from one another from week 6 onward. The “Trim” and “Trim+Late Spray” did not cause any significant change compared to the control group in terms of the proportion of colonized stems (Fig. 7A).

**Table 3.** Mixed model results for the 2019 and 2020 trimming + spray field plot experiments of proportion stems colonized by *E. giganteana*. **P < 0.01, ***P < 0.001

Model parameter	2019 Study (methoxyfenozide)			2020 Study (chlorantraniliprole)		
	*F*	df	*P*	*F*	df	*P*
Treatment	4.53	3,72	0.0057**	15.36	3,64	<0.0001***
Week	45.98	8,72	<0.0001***	58.96	7,64	<0.0001***
Treatment*Week	3.05	24,72	0.0001***	6.71	21,64	<0.0001***
Field covariance	0.0025			0.0000		
AR(1) covariance	0.9249			0.8506		
Residual covariance	0.0133			0.0074		

In 2020, when chlorantranililiprole was applied as the insecticide, there was again a significant week × treatment interaction (Table 3). Trimming alone did significantly reduce *E. giganteana* colonization early in the season, but beginning in mid-August, neither trimming alone nor Trim + Early spray had significantly lower colonization than the control treatments (Fig. 7B). The Trim + Late spray combination succeeded in significantly reducing *E. giganteana* colonization beginning week 4 (29 July, 10 days postapplication of chlorantraniliprole), and this reduction was maintained throughout the remainder of the sampling period (Fig. 7B).

## Discussion

The 4 experiments collectively demonstrated clear differences in insecticidal treatments on *E. giganteana* larvae. Neither *B. bassiana* nor Bt*k* was effective at inducing *E. giganteana* mortality or morbidity in the laboratory bioassay; additionally, Bt*k* did not reduce larva numbers in the field stems experiment.

While organic pyrethrins did not significantly increase death and morbidity of *E. giganteana* in the laboratory bioassay, synthetic pyrethroids did: β-cyfluthrin in the laboratory bioassay and permethrin in the field stems experiment reduced the number of live larvae. The effect of permethrin we observed was consistent with its effect on silflower capitula colonization observed in Vilela et al. (2020).

Nematodes were the only organic treatment in the laboratory bioassay that showed an arithmetically greater, though not statistically significant, effect on *E. giganteana* mortality and/or morbidity. Evidence suggests they are least effective as foliar treatments (Arthurs et al. 2004), therefore they were not applied as a foliar treatment in the stems or plot experiments. Given that *E. giganteana* overwinter underground, it would be useful to conduct future field studies investigating the efficacy of nematodes for *E. giganteana* management when applied as a soil drench.

The field stem study showed that neither bagging stems alone in late summer, nor bagging + Bt*k* applications, reduced larvae or proportion achenes damaged. In contrast, treating flower heads bagged or unbagged with methoxyfenozide and bagged flower heads with permethrin were effective. These results suggest that when larvae are already present, bagging alone may not reduce *E. giganteana* but spraying methoxyfenozide can. While we did not test the efficacy of permethrin in this study in the absence of bagging, Vilela et al. (2020) found that 0.4% permethrin applied to unbagged stems weekly for 3 wk reduced *E. giganteana* colonization of flower heads by 70%, 2 wk after the last application. The collective results show that these 2 insecticides could be effective control tactics for managing *E. giganteana* in individual stems when applied at higher concentrations. They should also be investigated for rotational use as part of an insecticide resistance management program.

The 2019 and 2020 trimming + insecticide experiments allowed us to evaluate the performance of 2 candidates – methoxyfenozide (Methoxyfenozide 2F) and chlorantraniliprole (Prevathon) – at a scale and application method more closely approximating an agricultural production setting. We observed that trimming alone only significantly delayed infestation in 2020, and in both years it was unable to significantly reduce the infestation through the end of the growing season compared to the untrimmed control. Moreover, its impact on crop yield is yet to be studied.

In 2019 methoxyfenozide only limited the *E. giganteana* population up to 1 mo prior to the usual harvest date (September–October), not for the entire growing season, when applied only once during the season (early or late spray) and paired with trimming. This differed from its efficacy to reduce *E. giganteana* infestation in the 2018 field stems experiment. This may be due to a difference in application rate. In the 2019 field experiment we followed the application rate for other Lepidopterans recommended by the manufacturer, which was 31% of the application rate that was applied in the 2018 field stems experiment. It is possible that *E. giganteana* may require higher or more frequent doses of this insecticide than was used in the 2019 field study if it were to be used commercially. A follow-up study is needed to determine if application rates may influence the performance of this insecticide.

Chlorantraniliprole was effective at reducing *E. giganteana* larvae in the laboratory and the field. More specifically, in the field 2020 trimming + insecticide experiment, a late application of chlorantraniliprole paired with trimming was the only tested treatment able to limit *E. giganteana* infestation during the entire season. That same experiment corroborates the previous assumption that trimming only postpones the infestation and does not alone suffice for pest control. However, further research is needed to assert whether chlorantraniliprole paired with trimming is more effective than chlorantraniliprole alone.

Regarding the effects of *E. giganteana* on silflower yield, our field stems study showed a direct correlation between the number of larvae per capitulum and seed damage, but it did not demonstrate that *E. giganteana* reduced yield. It is possible that either the tolerance of *S. intergrifolium* of *E. giganteana* is higher than the colonization rates we observed in this study, or that *E. giganteana* causes greater long-term yield loss when feeding on the root crowns than within-season yield loss when feeding on the flower heads. The disproportionate number of unfilled achenes may also contribute to this result. Potential reasons for low seed fill could be due to any 1 or a combination of the following factors: (i) Stems were harvested on 31 August, which is earlier than we normally harvest silflower seeds (usually Sept-Oct). Therefore, many of the “unfilled” achenes could be premature. (ii) The plants used in this study had previously been infested with *E giganteana* in their root crowns. Root crown feeding of *E. giganteana* may have deprived the plants of carbohydrate stores necessary for seed fill. (iii) The study plants were naturally infected with rust (*Puccinia silphii*, pers. obs.) which can have negative effects on silflower yield components such as leaf mass and flower head mass (Turner et al. 2018). However, published data do not yet exist on the specific effect of *P. silphii* on seed fill, nor did we collect data on the severity of rust infections.

The “Trim+Early Spray” outperformed other treatment in the early season when methoxyfenozide was applied, while “Trim+Late Spray” holds this place when chlorantraniliprole was applied in the 2019 and 2020 trimming + insecticide experiments, respectively. Apart from the dosage concerns previously discussed, alternative explanations for this difference in spray time efficacy include: (i) The 2019 and 2020 *E. giganteana* flights differed in both chronology and quantity of moths (Fig. 3), which may explain the fact that insecticidal sprays at similar dates had contrasting effects relative to the unsprayed control. (ii) The modes of action of methoxyfenozide and chlorantraniliprole differ, with methoxyfenozide disrupting molt in larvae by promoting premature apolysis and inhibiting ecdysis (Carlson et al. 2001), and chlorantraniliprole targeting specialized calcium release receptors in the endoplasmic reticula, causing insect muscle contraction paralysis and death (Troczka et al. 2017). Also, chlorantraniliprole is a systemic insecticide with translaminar activity (Pes et al. 2020), while methoxyfenozide is systemic only in roots and is not translaminar (Carlson et al. 2001). It is possible that *E. giganteana* has different levels of tolerance to insecticides with different modes of action.

Each trimming + insecticide field experiment only covered a single growing season. The *E. giganteana* population was lower in 2020 compared to 2019; therefore, the criterion used in the trimming + insecticide experiments to categorize a stem as colonized or not shifted between 2019 and 2020 (presence of a larva in the terminal flower head vs. presence of a larva on any flower head, respectively). Since we conducted the same trimming and control treatments within each year and analyzed the data for each year separately, it is unlikely the change in colonization criteria impacted the results. Additionally, the colonization rates in the control treatments in 2019 and 2020 were comparable despite the difference in both *E. gianteana* populations and colonization rate measurement criteria. However, it does limit the ability to quantitatively compare the efficacy of methoxyfenozide to chlorantraniliprole, as the 2 insecticides were applied under different field conditions. A follow-up field trial using different application rates and frequencies of both insecticides in the same season would provide a better comparison.

### Implications for *E. giganteana* Management in *S. integrifolium*

The de novo domestication of a novel crop inevitably comes with multiple challenges. One of the major challenges in domesticating silflower is the presence of a native specialist pest, *E. giganteana*. In this study we performed multiple small experiments to rapidly identify potential pest control solutions for *E. giganteana*. While the treatments we tested will require more studies to validate and fine-tune application rates and timing, this set of experiments nevertheless provided multiple options as a starting point for developing integrated pest management programs for *E. giganteana* in silflower as an oilseeds crop.

The most efficacious insecticidal treatments to control larvae were the pyrethroids (β-cyfluthrin and permethrin) and the diamide chlorantraniliprole. The insect growth regulator methoxyfenozide was also at least partially effective, in that it successfully reduced *E. giganteana* in the 2018 field stems study but not in the 2019 trimming + insecticide study. Since silflower is an obligate cross-pollinated species, determining which of these or similar insecticides should be incorporated into an IPM program will depend in part upon the insecticides’ effects on pollinators and seed set. Multiple studies have demonstrated that pyrethroids are toxic to pollinators (Oliver et al. 2015, Piccolomini et al. 2018, [Bibr CIT0022][Bibr CIT0008]). Therefore, the timing and frequency of pyrethroids, if used, must be balanced with the needs of its necessary pollinators.

Methoxyfenozide, while generally considered less toxic to pollinators than pyrethroids, nevertheless can negatively affect colony survival and behavior of *Apis mellifera* (Hymenoptera: Apidae) (Fisher et al. 2018, Meikle et al. 2019). Chlorantraniliprole has not been shown to negatively affect honeybees when applied at agronomic rates (Williams et al. 2020), but can have sublethal effects on behavior and reproduction in *Bombus terrestris* (Hymenoptera: Apidae) (Smagghe et al. 2013). While these 2 insecticides still appear to be more pollinator-friendly treatment options than many conventional pesticides, follow-up research is needed to determine the application rates, application timing, and number of applications of these insecticides that can effectively reduce *E. giganteana* while minimizing negative effects on pollinators and, subsequently, silflower pollination.

Trimming plants as a mechanical tactic to delay flowering showed partial success in reducing *E. giganteana* colonization in early summer 2020, when *E. giganteana* populations were relatively low. However, it did not reduce colonization significantly at any point in 2019 (consistent with the results from Vilela et al. 2020) or in the latter part of the 2020 growing season. Collectively these studies suggest that trimming alone does not suffice as a cultural control tactic for *E. giganteana.* However, trimming when paired with a later treatment of chlorantraniliprole could be an effective tactic as part of an IPM program for *E. giganteana*. The yet unknown aspect of this strategy is whether trimming the top 15% of the plants just prior to flowering significantly affects crop yield later in the season.

Of the techniques we attempted, there were a variety of treatments identified that could be used to manage *E. giganteana* in silflower. Nonetheless, further research will be needed to expand this work into a useful IPM program at the agronomic production level. Future work will also be needed to establish how *E. giganteana*, and the mitigation strategies to manage it, affect plant health and yield in silflower.
